# A New Curb Detection Method for Unmanned Ground Vehicles Using 2D Sequential Laser Data

**DOI:** 10.3390/s130101102

**Published:** 2013-01-16

**Authors:** Zhao Liu, Jinling Wang, Daxue Liu

**Affiliations:** 1 College of Mechatronics & Automation, National University of Defense Technology, Changsha 410073, Hunan, China; E-Mail: daxue_l@yahoo.com.cn; 2 School of Surveying and Geospatial Engineering, the University of New South Wales, Sydney 2052, Australia; E-Mail: jinling.wang@unsw.edu.au

**Keywords:** curb detection, laser range finder, mapping, dynamic environment

## Abstract

Curb detection is an important research topic in environment perception, which is an essential part of unmanned ground vehicle (UGV) operations. In this paper, a new curb detection method using a 2D laser range finder in a semi-structured environment is presented. In the proposed method, firstly, a local Digital Elevation Map (DEM) is built using 2D sequential laser rangefinder data and vehicle state data in a dynamic environment and a probabilistic moving object deletion approach is proposed to cope with the effect of moving objects. Secondly, the curb candidate points are extracted based on the moving direction of the vehicle in the local DEM. Finally, the straight and curved curbs are detected by the Hough transform and the multi-model RANSAC algorithm, respectively. The proposed method can detect the curbs robustly in both static and typical dynamic environments. The proposed method has been verified in real vehicle experiments.

## 1. Introduction

Environment perception is a key research direction in the area of UGV development. The UGV is expected to navigate autonomously in semi-structured environments such as campus sites, parks, and the urban environment. It is important for an UGV to be able to detect obstacles around it correctly in order to avoid the risks of collision. The road curb is a special sub-category that can represent the boundary of the road so as to calculate the obstacle-free areas.

According to the different features of the curb detection, the existing algorithms are divided into two categories: the first is based on detection of the geometrical features of the curb; the second category is based on image context derived from the monocular vision field. The main merit of the second method is that it contains rich information, such as color, texture, *etc.* Commercial lane departure warning systems based on monocular vision are available now [1,2]. However, the method belongs to the first category which has problems to cope with bad situations such as poor illumination, bad weather and insufficient road lanes. Compared with the second category, in general, the first category is not affected by the above factors, except stereo vision, and is adaptive and robust to the varied environment. Therefore, we will concentrate on the first category of algorithms in this research.

The geometrical features of a curb are not clear in a real structural environment, as curb height varies only from 5 cm to 25 cm in general. Therefore, curb detection is a challenging task, because the geometrical features of a curb might be contaminated by random noise and measurement errors. In recent years, researchers have presented some curb detection algorithms using geometrical features which are obtained by laser range finder [3–8], stereo vision [9–11], TOF Camera [12,13] and structure light [14]. According to the idea of curb detection, such algorithms can be divided into two classes: one idea is that range data is processed individually per frame and the algorithm can obtain curb candidate points, then the curb points are tracked using the filter method, such as Kalman filter and Extend Kalman filter; the other idea is that the algorithm extracts ground surface or obstacle-free area in a local map to obtain local curb information.

In [3,4], Kodagoda *et al.* used a tilted 2D laser range finder to detect road curbs. In this approach, the result of the measurement is predicted with the Kalman filter algorithm. If the measurement is far away from the prediction, it is considered as a curb candidate. Prior knowledge assumptions have to be made to find the right curbs. For example, the UGV needs to move in parallel to the curb, the street width is known, and the curbs are locally straight. Furthermore, Kodagoda *et al.* proposed an effective curb tracking and estimation method with camera and laser range finder data in [5]. Smadja *et al.* [6] also presented a curb detection approach using a 2D laser range finder. Firstly, the road surface points are extracted by the RANSAC algorithm and are projected onto a global map; secondly, the boundaries of the obstacle-free area are fitted by the RANSAC algorithm, and then the curb candidate points are picked out by a multi-frame accumulated map. In [7], the author detected road curbs using HDL64-E LIDAR which contains 64 scan lines, but the algorithm deals with the data to be one line as a unit. Zhang proposed a road boundary method which are extracted using the elevation information based on the single data frame from the 2D laser range finder in [8], and the method had been verified in the 2007 DARPA Urban Challenge. The ideas of the above curb algorithms belong to the first class algorithms based on geometrical features.

In [9,11], the stereo vision is used for detecting curbs. A straight curb is detected by the Hough transform, and a curved curb is extracted by chains of segments in [9]. In [11], the author changed the curbs model to cubic polynomial curves, and then used the RANSAC algorithm to compute the parameters of the model. The authors built a DEM to detect curbs by Conditional Random Field (CRF) in [10]. The approach can detect and reconstruct different curvature and height curbs, but the algorithm assumes that the curb is visible in front of the vehicle. If the curb is occluded by another object on the road, the performance of the algorithm will be decrease. Gallo *et al.* [12,13] proposed a modified RANSAC algorithm (dubbed CC-RANSAC) for detecting street surface and pavement surface using a Canesta TOF Camera. The algorithm can detect two nearby surfaces by the largest connected components of inliers. The above approaches [9–13] belong to the second class algorithms based on geometrical feature.

In this paper, we propose a new curb detection method based on a local DEM which is built from 2D sequential laser range finder data and vehicle state data. The algorithm can detect road curbs accurately and quickly in both static and typical dynamic environments where a few objects may move on the road or roadside, and it belongs to the second class based on geometrical feature algorithms. Compared with the first class algorithms, our method has three main merits. Firstly, our method can obtain robust curb detection results, because the historical and current sensor information is considered in the process of the curb detection. To be precise, our method uses the local DEM information which includes multiple laser data frames to detect the curb. The first class algorithms only use limited information, so these algorithms are sensitive to data noise. Secondly, we only select the suitable curb model in our method, and the curb tracking step is not available. The reason is that the curb model implies knowledge of the geometric information of the road. If we obtain the parameters of the curb model, we will not need to track the new curb. However, curb tracking is an important step after the curb detection in the first class algorithms, because it can check the validation of the new result based on the filter which includes the former curb information. In general, curb tracking is a difficult task using the traditional tracking methods such as the Kalman filter. The main reason is that the traditional tracking methods need to know the accurate process model and estimate the error model, which are hard to obtain in practice. Thirdly, our method can detect the curbs in a typical dynamic environment, which reduces the influence of moving objects in the process of the building the local DEM.

The paper is structured as follows: in Section 2, the proposed method is described, and the curb detection algorithm will be presented; the experimental results are shown in Section 3 and conclusions in Section 4.

## 2. Design of the New Curb Detection Method

### 2.1. The Overview of the Method

**Figure 1.**
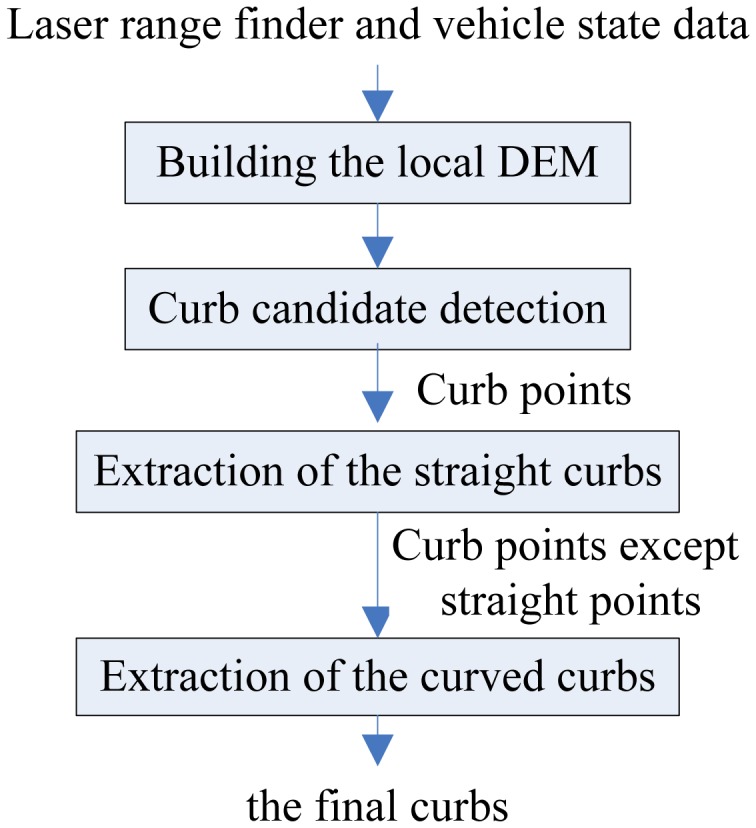
Flowchart of the new curb detection method.

The basic idea of the proposed curb detection method based on the geometrical features are mentioned in Section 1. There are four steps in the proposed new method. The schematic of the new curb detection method is shown in Figure 1.

First, a local DEM is built in real-time by a 2D laser range finder and vehicle state data which denotes the surrounding environment information of the vehicle. Second, curb candidate points are extracted in the local DEM and we accumulate the multiple results of curb candidate points. Third, the straight curbs are obtained by the Hough transform algorithm and some constraint conditions in the accumulated curb candidates. Then, the residual curb candidate points, except straight curbs, are processed by the multi-model RANSAC algorithm which uses the suitable model to represent the curved curb. Each step is discussed in detail in the following.

### 2.2. Building a Local DEM

#### 2.2.1. Building the DEM in a Static Environment

The construction of models of the environment is crucial in UGV operations. There are many environment models, such as elevation grids, point clouds, 3-D grids, and meshes [15]. In this research, the elevation grid model is chosen to represent the surrounding environment of a UGV. This has two main merits. First of all, though the representing environment functionality of the elevation grids is weaker than point clouds, 3-D grids, and meshes, the algorithm of building the environment model has a better real-time performance than other environment representation models. Secondly, the elevation grids can accumulate many data frames from the 2D laser range finder. In other words, it promotes the performance of the 2D laser range finder which obtains only one scan line per frame and offers more abundant environment information, so the curb detection algorithms based on elevation grids have better reliability and accuracy. We build the local DEM using 2D sequential laser rangefinder data and vehicle state data which contains global position and altitude information. Our algorithm detects the road curb based on the local DEM in the global coordinate system.

**Figure 2.**
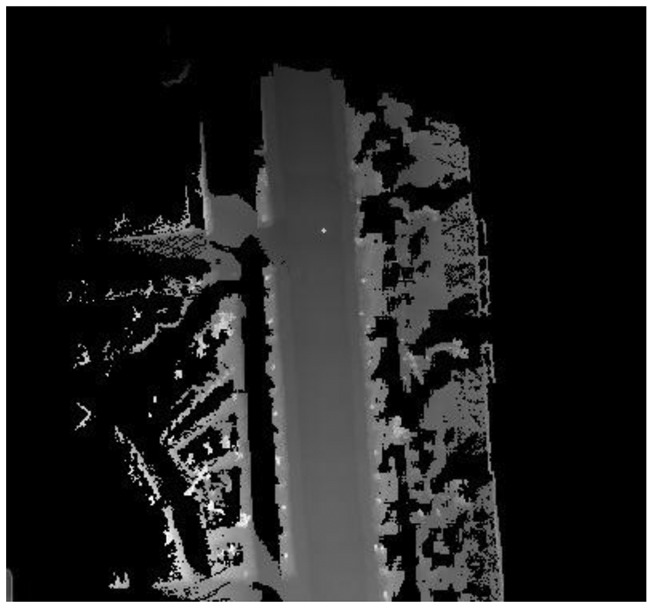
The local DEM in static environment.

According to environmental complexity, vehicle navigation requirement and computing power of the system, we build an 80 × 80 m local DEM, and the grid size is 20 × 20 cm. Figure 2 shows an example of the local DEM in the static environment. The gray level denotes the height of the terrain, and the larger gray level represents the higher height. The position of vehicle is denoted by a yellow point in Figure 2 which located in the center area of the map. The road region and roadside can be distinguished in Figure 2. We can find that the local DEM can represent the surrounding environment of a vehicle accurately in the static environment. Unfortunately, real world environments are dynamic rather than static. Moving objects which include vehicles, pedestrian, bicycles and so on usually appear in the environment, so we must consider the impact of moving objects in the process of building the local DEM. Next section we will describe the mapping process in a dynamic environment.

#### 2.2.2. Building the DEM in a Dynamic Environment

**Figure 3.**
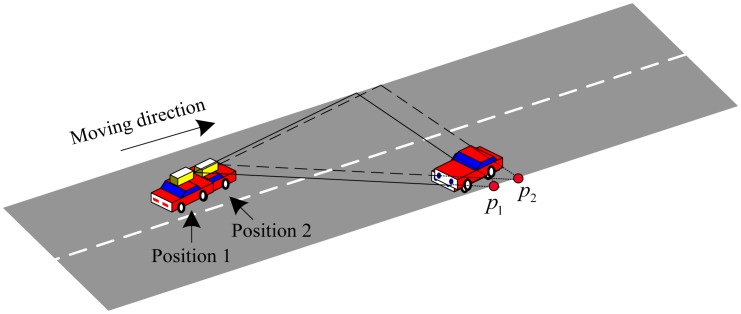
The schematic of the laser scanning on the road.

**Figure 4.**
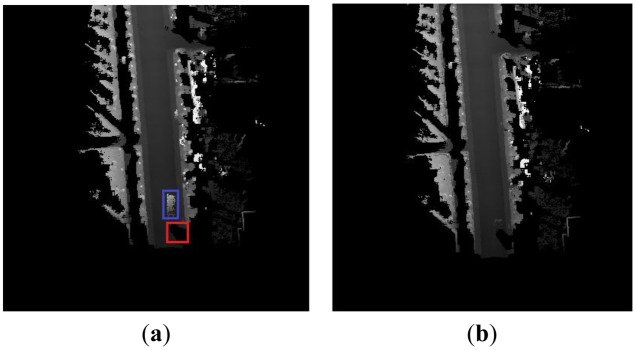
The local DEM in dynamic environment. (**a**) The bad result. (**b**) The result of our approach.

Building the DEM is a challenge task with 2D laser range finder in a dynamic environment. We not only have to determine the position of the vehicle and the map of the environment, but also identify and deal with possible moving objects in the environment. Compared with the static environment, there are some difficulties of building the DEM in a dynamic environment. The first one is that some spurious objects appear in the passing area of moving objects. The phenomenon is shown in the blue rectangle areas of this Section. The long white trace is a spurious object in blue rectangle areas which will have a serious influence on the curb detection. The second one is that the occlusion problem that happens more frequently in a dynamic environment than that in a static environment. This will cause missing data for some parts of the curb information in the DEM. The missing data cannot be obtained again by the 2D laser range finder, because of the scan principle and the way of installation of the 2D laser range finder which is shown in Figure 3. We can see that the laser range finder of the left vehicle cannot detect the curb point *p*_1_ in position 1 in Figure 3, because it is occluded by the right vehicle. If the left car moves on the road from position 1 to position 2, the curb point *p*_2_ cannot be detected because of the occlusion of the right vehicle. The curb point *p*_1_ cannot be detected even during this moving process because the 2D laser range finder has only one scanning plane which is shown in Figure 3 where two triangles areas consist of the dash lines and the solid lines independently. In other words, if the laser range finder misses the curb point *p*_1_ in position 1, it has no chance to scan *p*_1_ again. The occlusion can bring about difficulties in the curb detection. The situation of the missing data appears in the red rectangle areas due to the occlusion of the moving object in Figure 4(a).

A probabilistic moving objects deletion approach is proposed in this paper which can delete most of spurious objects caused by moving objects in the process of building the DEM. The purpose of the approach is to decrease the influence of moving objects in the DEM and to keep the geometric feature of the road areas which are the passing areas of moving objects at the same time. In other words, we want to build the static environment map in the dynamic environment. The main idea behind the proposed method is to use the time cue based on the probability approach to detect the data of the moving objects.

We define the function which is called data permit mapping probability function (DPMPF). Firstly, the DPMPF of the each cell is initialized: *P_i,j_*(Δ*t*) = 1, where *P_i,j_*(Δ*t*) denotes the data permit mapping probability in each cell. Secondly, If a new measurement point appears in *cell*(*i*,*j*), the *P_i,j_*(Δ*t*) will be updated by the following formulation:

(1) $$P_{i,j}(\Delta t)=\{\begin{array}{ll} P_{i,j}(\Delta t) & 0<\Delta t\leq 0.015\\ P_{i,j}(\Delta t)-\Delta t & 0.015<\Delta t \end{array}$$

where Δ*t* = *t_new_* − *t_last_*, Δ*t* denotes the time interval from the last laser measurement *t_last_* to the new laser measurement *t_new_* in *cell* (*i*,*j*). The threshold (0.015 s) is chosen by the scan frequency of the laser range finder. *t_last_* is updated by the following formulation:

(2) $$t_{\text{last}}=\{\begin{array}{cc} t_{\text{new}} & P_{i,j}\;(\Delta t)>T_{1}\\ t_{\text{last}} & P_{i,j}\;(\Delta t)\leq T_{1} \end{array}$$

where *T*_1_ is the threshold, and is defined by the prior knowledge. Here, we choose *T*_1_ = 0.5 s. If *t_last_* is updated, the new measurement will be mapped in the DEM. Namely, the new measurement does not belong to the part of a moving object. Figure 4 shows an example of the local DEM in a dynamic environment. Figure 4(a) represents a bad DEM, which does not deal with the moving object in the process of building a DEM. The DEM built by our new method which deletes the spurious object is shown in Figure 4(b). We can see that most of the spurious object is deleted, and the remainder of the spurious object is small in Figure 4(b). Our approach can reduce the interference of a moving object and maintain the geometric information of the road area. This is very essential for the subsequent curb detection.

### 2.3. Curb Candidate Extraction

In our method, it is assumed that the height of the ground surface varies continuously and slowly. The curb candidate point has a main feature: elevation gradient variation in the local DEM. We design a curb candidate point detection algorithm based on the vehicle's direction of movement. The algorithm assumes that the vehicle is located on the road surface, and chooses the appropriate direction to detect the elevation gradient variation in the adjacent grids. The above detected directions of the grid depend on the vehicle moving direction, but not equal to the vehicle moving direction. The curb candidate point should meet the needs of the following conditions:

- The slope between the curb candidate grid (point) and the adjacent grids is large enough. The formula for slope calculation is as follows:
  (3) $$\tan\theta =\frac{z_{1}-z_{2}}{\sqrt{{\left(x_{1}-x_{2}\right)}^{2}+{\left(y_{1}-y_{2}\right)}^{2}}}$$
  where (*x*_1_, *y*_1_) and (*x*_2_, *y*_2_) denote grid coordinates in the local DEM; *z*_1_ and *z*_2_ are the height of the grid.
- The height difference which is denoted Δ*h_1_* in a same curb candidate grid is larger than a given threshold *T*_2_.
- The height variance Δ*h_2_* between the curb candidate grid and the adjacent grid meet the following formula:
  (4) $$h_{\min}\leq \Delta h_{2}\leq h_{\max}$$
  where *h*_min_ denotes the lower limit of height variance, *h*_max_ denotes the upper limit of height variance.

**Figure 5.**
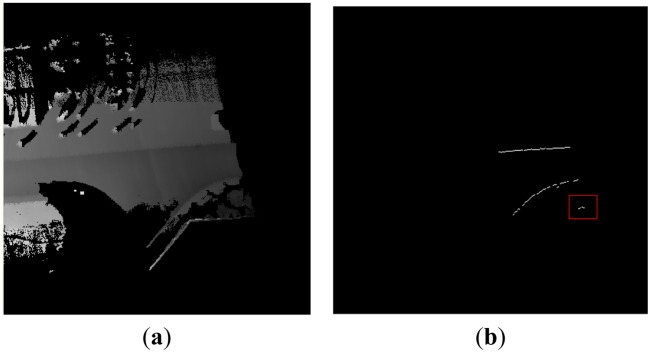
The results of the curb candidate detection. (**a**) The local DEM. (**b**) The curb candidate points in the local DEM.

The results of the curb candidate detection are shown in Figure 5. Figure 5(a) represents a current local DEM around the vehicle. The result of curb candidates is shown in Figure 5(b), in which the white points denote curb candidate points. We can find that our algorithm can detect the straight and curved curb candidate points.

**Figure 6.**
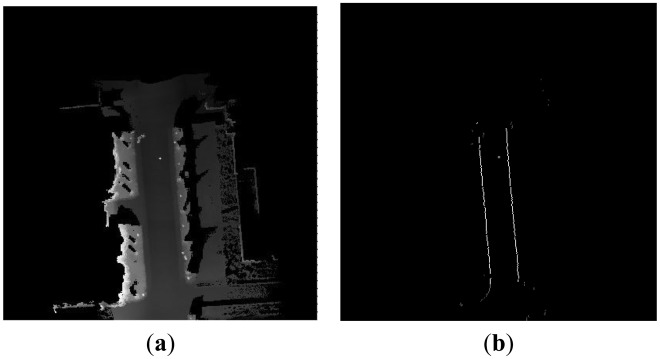
The accumulated results of the curb candidates. (**a**) The local DEM. (**b**) The accumulated curb candidate points in the local DEM.

However some false candidate points arise in the red rectangle area in Figure 5(b). There are two reasons for this error. Firstly, due to the obstacle occlusion on the road, the DEM does not reflect the true geometry structure of road surface and curb. Secondly, the geometrical feature of a curb is not obvious, so the curb is easily contaminated by random noise and measurement errors. Because of the above reasons, the result of the single curb candidate detection is not reliable. We accumulate the multiple results of curb candidate points to improve the accuracy of the detection algorithm. In the local DEM, we set a counter in each grid. The counter will record the number of the curb candidate points which appear in the same grid. If the number is larger than a given threshold, the point will be used in the next step. The valid curb candidate point will be kept in the local DEM. Figure 6(a) represents a local DEM around the vehicle. The gray points denote the accumulated points of the curb candidate in Figure 6(b). The accumulated points record the historical curb information in the rear of the vehicle.

Note that the results of the curb candidate detection only have the right part in Figure 5(b). The reason is that the detected area from our algorithm is influenced by the current vehicle position and direction of movement. The direction of movement of the vehicle is approximately towards the East, so the right parts of Figure 5(a) are detected. In the following section, the same problem will be encountered, and will not be explained again.

### 2.4. Curb Detection

In this section, the real road curbs are extracted from the result of the accumulated curb candidates. The curbs are divided into two classes: straight and curved curbs. According to the different characteristics of the straight and curved curbs, we use the Hough transform to extract the straight curbs and the multi-model RANSAC algorithm to extract the curved curbs.

#### 2.4.1. Extraction of Straight Curbs Based on the Hough Transform and Multiple Constraints

Firstly, the candidate straight curb is detected by the Hough transform after the accumulated results of the curb candidates are handled by the isolated point filter algorithm to eliminate random noise. There are two reasons to adopt the Hough transform to detect the straight curbs. The first reason is that the Hough transform has a good adaption to a noisy environment. Compared with it, the traditional method such as the least squares can be easily affected by gross errors, leading to wrong results. The second reason is that the Hough transform considers the entire distribution of the data set, so it can give more accurate result than the incremental line algorithms which use the local data distribution. Although the processing time of the Hough transform is longer than the least squares algorithm and incremental algorithms, it still satisfies the real-time operation requirement. The average and the worst computational time of our algorithm are about 2.45 ms and 4.91 ms, respectively.

Secondly, the real straight curb is selected from the candidate straight curbs based on the results of the Hough transform. We have designed a two-stage scheme to choose the best straight curb: (1) the candidate straight curbs are divided into three categories: the left straight curbs, the right straight curbs and other curbs according to the position and direction of the vehicle; (2) we will use three constraints to choose the best straight curbs in the first two categories. Based on the above classification, we have designed the following constraint conditions:

- The direction constraint:
  (5) $$\begin{array}{cc} \left|\phi_{c}-\theta_{i}\right|<\delta_{1} & 0<i<n \end{array}$$
  where *ϕ_c_* denotes the current yaw angle; *è_i_* denotes the angle between the x axis and the candidate straight curbs; *n* denotes the number of candidate straight curbs; *δ*_1_ denotes an angle threshold.
- The constraint of the historical straight curb information:
  (6) $$\begin{array}{cc} \left|\theta_{\text{old}}-\theta_{i}\right|<\delta_{2} & 0<i<n \end{array}$$
  where *è_old_* denotes the historical angle between the *x* axis and the old candidate straight curbs; *δ*_2_ denotes an angle threshold. It is assumed that the straight curbs vary regularly and continuously.
- The life cycle constraint:
  (7) $$t_{\text{new}}-t_{\text{old}}<T_{1}$$
  where *t_new_* denotes the time of the new detected straight curb; *t_old_* denotes the time of the last (historical) detected straight curb; *T*_1_ denotes the life cycle. The constraint means that the validation of the historical straight curb information is restricted in our algorithm. In other words, the historical straight curb information has a life cycle. If *t_new_* – *t_old_ T*_1_, the historical straight curb information will be invalid.

#### 2.4.2. Extraction of the Curved Curb Based on the Multi-Model RANSAC Algorithm

The curved curb detection is an important research area, because curved curbs usually appear in practice. In this part, the multi-model RANSAC algorithm is proposed to detect the curved curbs. RANSAC [16] was proposed by Fischler and Bolles in 1981. The merit of RANSAC is its ability to perform robust estimation of the model parameters, particularly when a significant percentage of data are outliers, but the RANSAC algorithm has two drawbacks. The first one is that it requires the user to choose the suitable model according to the demand of the specific problem. If the user chooses the unsuitable model, RANSAC algorithm will give a wrong result. The second one is that RANSAC can only estimate one model for a data set. When two (or more) model instances exist, the RANSAC may fail to find either one.

**Figure 7.**
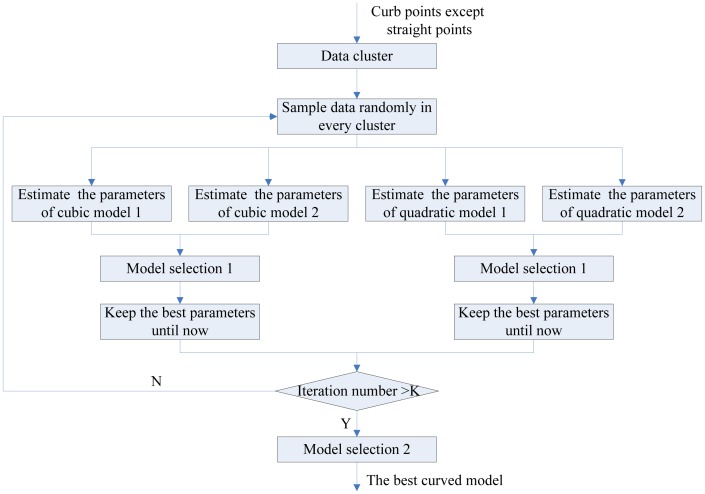
The flowchart of the multi-model RANSAC algorithm.

The ordinary RANSAC algorithm is unsuitable for extraction of the curved curb because of the above drawbacks. There are two reasons. The first one is that the number of the curved curbs is equal or greater than one when the curb exists. The second one is that the shape of the curved road is complex. Namely, with a one curved curb model it is hard to describe the entire curved road. In this paper, the multi-model RANSAC algorithm is proposed to deal with the above problems. The merit of our algorithm is that it can cope with multiple models at the same time and select a suitable curved curb model. The flowchart of our algorithm is shown in Figure 7. Firstly, the accumulated curb candidate points are clustered by a distance criterion. Secondly, some preliminary curved curb models are estimated and our adaptive model selection step is used to obtain the most suitable curved curb model in each cluster with a certain number of the points.

There are two purposes of setting up a data cluster. The first purpose is that the multiple curved curb candidates are divided into different clusters before fitting the parameters of the model; the second purpose is to reduce the noise points in the data set.

According to the complexity of curbs in the real road environment, we have selected the quadratic polynomial model and cubic polynomial model to represent the curved curb. The adaptive model selection step can choose the suitable curved curb model online from these models in our algorithm. Formally, the four models are split into two groups as follows:

(8) $$\{\begin{array}{c} y=a_{0}\cdot x^{3}+a_{1}\cdot x^{2}+a_{2}\cdot x+a_{3}\\ x=b_{0}\cdot y^{3}+b_{1}\cdot y^{2}+b_{2}\cdot y+b_{3} \end{array}$$

(9) $$\{\begin{array}{c} y=c_{0}\cdot x^{2}+c_{1}\cdot x+c_{2}\\ x=d_{0}\cdot y^{2}+d_{1}\cdot y+d_{2} \end{array}$$

where the parameters *a*_0_, *a*_1_, *a*_1_, *a*_3_ and *b*_0_, *b*_1_, *b*_2_, *b*_3_ denote the coefficients of cubic polynomial models individually; the parameters *c*_0_, *c*_1_, *c*_2_ and *d*_0_, *d*_1_, *d*_2_ denote the coefficients of quadratic polynomial models individually. Note that only one model is valid when we use it to a sample from the data set.

In our algorithm, the adaptive model selection step includes two parts in Figure 7. The first part is that the good quadratic polynomial model and cubic polynomial model are selected individually in the same order model. The condition of the model selection is the percentage of the inliers in a cluster. The output model should have the maximum percentage of inliers. The second part is to select the best curved curb model in a different order model. The residual error of the model is used to select the best model. Here, the residual error of the model is defined as follows:

(10) $$e_{k}=\sum_{i=1}^{m}{\left(y_{i}-{\tilde{f}}_{k}(x_{i})\right)}^{2}$$

where *f̃*(●) denotes the estimated curved curb model; *k* denotes the order of the model; (*x_i_*, *y_i_*) denotes the data point of the cluster; *m* denotes the number of the data points. We define $e_{k}^{\text{inliers}}$ which denotes the residual error of the inliers. There are three rules of model selection:

- *if* $(e_{2}^{\text{inliers}}-e_{2}^{\text{inliers}})(e_{2}-e_{3})>0$
  (11) $$\text{best model}=\{\begin{array}{ll} \text{quadratic} & \text{if}e_{2}-e_{3}\leq 0\\ \text{cubic} & \text{if}e_{2}-e_{3}>0 \end{array}$$
- *if* $(e_{2}^{\text{inliers}}-e_{2}^{\text{inliers}})(e_{2}-e_{3})=0$
  (12) $$\text{best model}=\{\begin{array}{ll} \text{quadratic} & \text{if}e_{2}-e_{3}\leq 0\;\text{or}\;e_{2}^{\text{inliers}}-e_{3}^{\text{inliers}}\leq 0\\ \text{cubic} & \text{if}e_{2}-e_{3}>0\;\text{or}\;e_{2}^{\text{inliers}}-e_{3}^{\text{inliers}}>0 \end{array}$$
- *if* $(e_{2}^{\text{inliers}}-e_{2}^{\text{inliers}})(e_{2}-e_{3})<0$
  (13) $$\text{best model}=\{\begin{array}{ll} \text{quadratic} & \text{if}e_{2}-e_{3}-(e_{2}^{\text{inliers}}-e_{3}^{\text{inliers}})\leq 0\\ \text{cubic} & \text{if}e_{2}-e_{3}-(e_{2}^{\text{inliers}}-e_{3}^{\text{inliers}})>0 \end{array}$$

**Figure 8.**
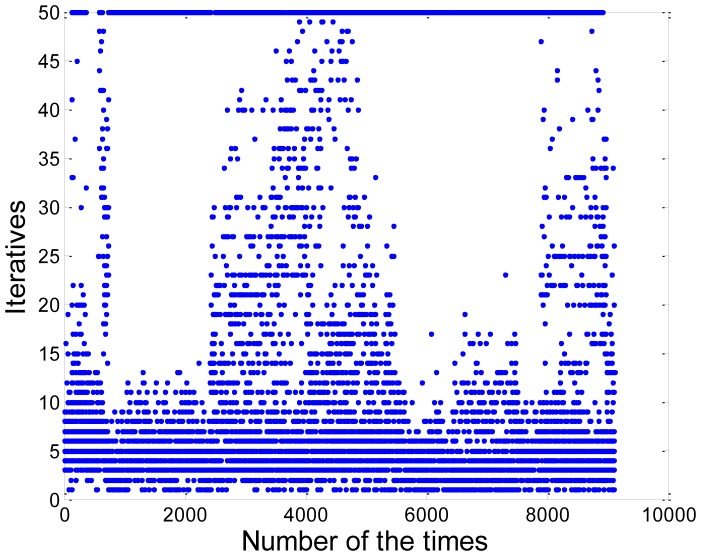
The iterative number of the RANSAC algorithm.

In order to increase the efficiency of our algorithm, we evaluate and modify the iterations which denotes *k*. The formula is shown as follows [17]:

(14) $$k=\frac{\log(1-p)}{\log(1-w^{n})}$$

where *p* denotes the probability that the algorithm produces a useful result. In other words, the algorithm selects *n* points of which all are inliers; *w* denotes the probability of choosing an inlier each time, and the formula as follows:

(15) $$w=\frac{n_{\text{inliers}}}{n_{\text{all}}}$$

where *n_inliers_* denotes the number of inliers in the data; *n_all_* denotes the number of points in the data. Assume that the *n* points which are selected independently are needed for estimating a model. *w^n^* is the probability that all *n* points are inliers and 1 − *w^n^* is the probability that at least one of the *n* points is an outlier, a case which implies that a bad model will be estimated from this point set. In general, the parameters *p* and *n* are constant if the model and data sets have been selected. Here, we set *p* = 0.99, *n* = 3. But *w* is unknown in advance, so *w* is estimated in the iterative process of the algorithm. According to the above formula, *k* can be estimated and the algorithm can choose the appropriate number of iterations and enhance the efficiency. The estimated results of the parameter *k* are shown in Figure 8. Here, we choose the initial iterations *k*_0_ = 50. We can see that the majority of the iterations less than 20, and only a few iterations close to *k*_0_. According to our calculation, in comparison with the original algorithm, the efficiency of the multi-model RANSAC algorithm has been increased by 47.64%. The average and the worst computational time of our algorithm are about 2.44 ms and 24.89 ms, respectively. The details of the computer and software are introduced in Section 3.

## 3. Experiments

**Figure 9.**
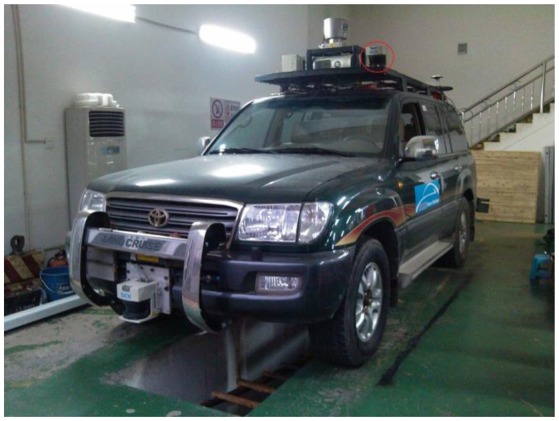
The position of the laser range finder.

The experimental platform is a light off-road vehicle. A forward-looking laser range finder is mounted on the front top of the vehicle and tilts down a little, and it is shown in the red ellipse area in Figure 9. The LMS-291 SICK sensor and SPAN-CPT integrated navigation system produced by the NovAtel Company were used in the experiment. The LMS-291 scans 180° field-of-view with 1° resolution at a 75 Hz scan rate, and the work mode is the interlace mode [18]. It was installed at the height of 2 m to scan the road surface about 17 m ahead. The SPAN-CPT system used the DGPS mode in the experiment. The experiments were done on our campus. There are two experiments in this part. The first experiment is to verify the effectiveness of our algorithm. The second experiment is to analyze the results of our algorithm quantitatively.

**Figure 10.**
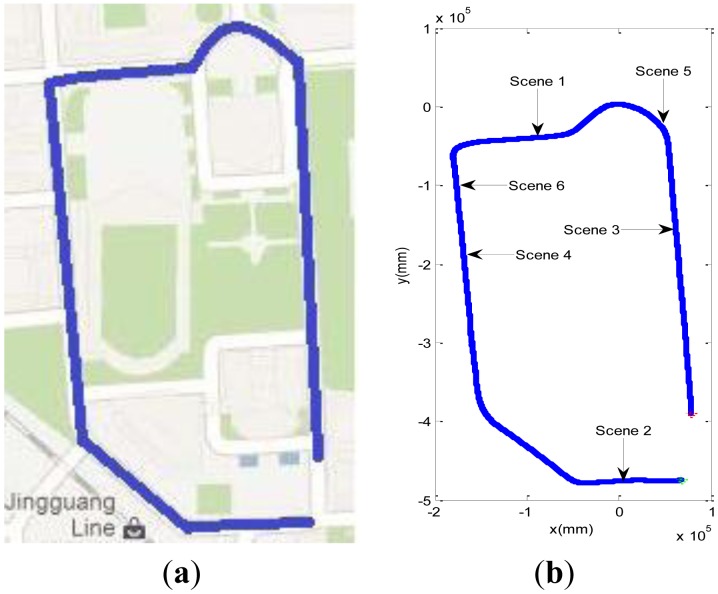
The running route of the vehicle in first experiment. (**a**) The experimental site in the Google map. (**b**) The position of four scenes.

The first experimental site is shown in Figure 10(a). The blue lines denote the route of the vehicle in Figure 10, which are recorded from the navigation system of our vehicle. The travelled distance of the vehicle is 1.6 km. We select four typical scenes from the whole data set, and the positions of these four scenes are shown in Figure 10(b). The red point denotes the start point of the vehicle, and the green point denotes the end point of the vehicle. The experimental results of six scenes will be introduced respectively in the following.

**Figure 11.**
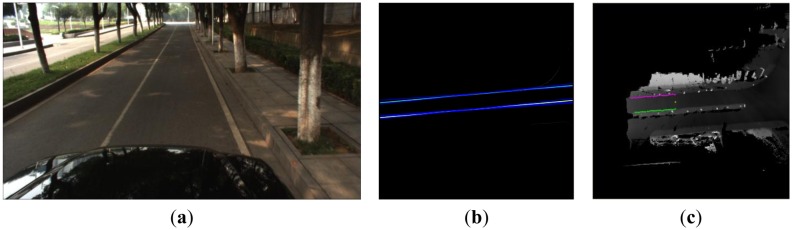
The result of the straight curb detection. (**a**) Scene 1. (**b**) The results of Hough transform. (**c**) The final curb detection results in the local DEM.

Typical straight curbs exist in Scene 1, and the vehicle drove from the East to the West. Figure 11(a) presents the corresponding visual image in front of the vehicle and the two obvious straight curbs were seen in Figure 11(a). The line detection result using the Hough transform is shown in Figure 11(b). There are four lines in Figure 11(b), the two white lines are selected as the results according to the constraint condition in Section 3.2.1. Figure 11(c) shows the final straight curb result. The yellow points denote the position of the vehicle, the green points denote the detected left curb points and the pink points denote the detected right curb points. Our algorithm can detect the straight curb on the road accurately.

**Figure 12.**
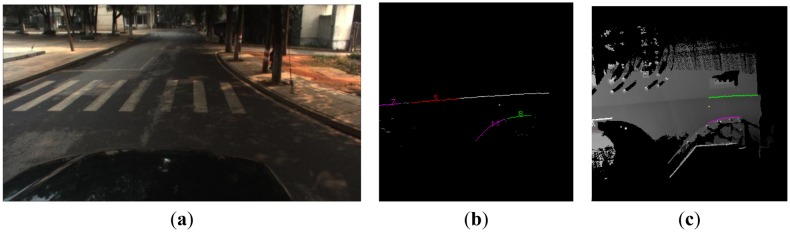
The result of the curb detection. (**a**) Scene 2. (**b**) The data cluster results of the curb candidate points. (**c**) The final curb detection results in the local DEM.

In Scene 2, the right curved curb and the left straight curb were seen in Figure 12(a), and the vehicle drove from the West to the East. The data cluster result is shown in Figure 12(b). The different colored points represent the different classes which not include the whole cluster result and only useful classes except the gray points which are unclassified. Figure 12(c) shows the final curb detection result. The right curved curb is detected by our curved curb algorithm, and the left straight curb is detected by our straight curb algorithm. It has been verified that the proposed algorithm can detect curved curbs successfully.

**Figure 13.**
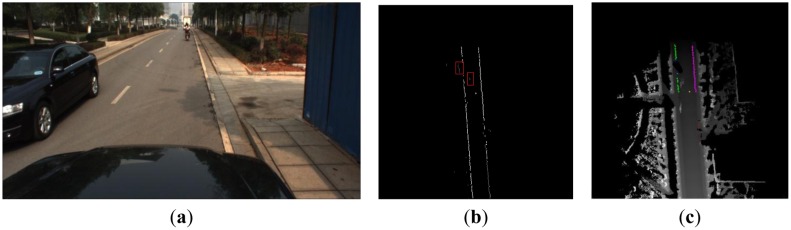
The curb detection in typical dynamic environment. (**a**) Scene 3. (**b**) The accumulated results of the curb candidates. (**c**) The final curb detection results in the local DEM.

Scene 3 is a typical dynamic environment, and vehicle can be seen on the road in Figure 13(a). Because the moving vehicle partly occluded the left road curb, missing data appeared in Figure 13(c). In Figure 13(b), the two red rectangles are some wrong results in the result of accumulated curb candidates. The left wrong result was caused by the missing data so the algorithm actually detected the roadside vegetation. The right wrong result was caused by the process of building the DEM due to the interference of the moving vehicle. In this situation, the difficulty of the curb detection increased, but the proposed algorithm can detect the left straight curb except for the region of the missing data.

**Figure 14.**
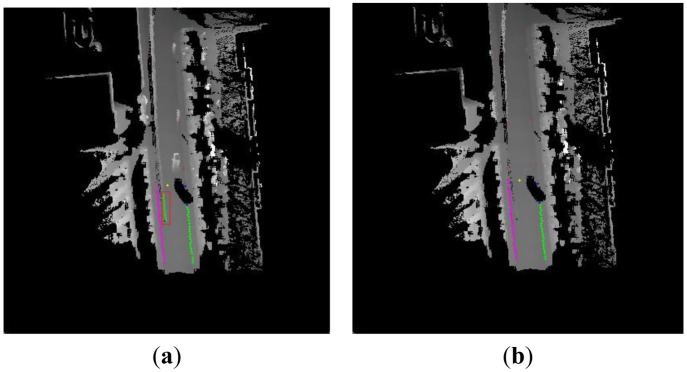
The contrastive result of the curb detection. (**a**) The bad curb detection result. (**b**) The result of our method.

Scene 4 is a typical dynamic environment too, but it is more complex than Scene 3. Some dynamic objects which include the car and the bicycles appear in the local DEM in Figure 14(a). Because of this, a lot of spurious objects appeared in the local DEM in Figure 14(a), because the effect of the moving objects is not considered. We can find that the obvious error of curb detection appears in red rectangle area in Figure 14(a). The above error arises from the spurious objects. The local DEM was built by our approach in Figure 14(b), and the spurious objects are decreased, so we obtain a good curb detection result. All in all, our proposed algorithm can reduce the effect of moving objects and build a good quality DEM for use in detecting the curbs.

**Figure 15.**
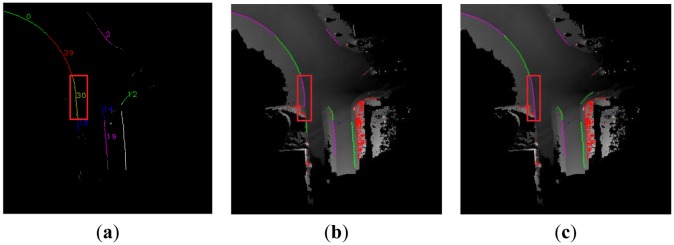

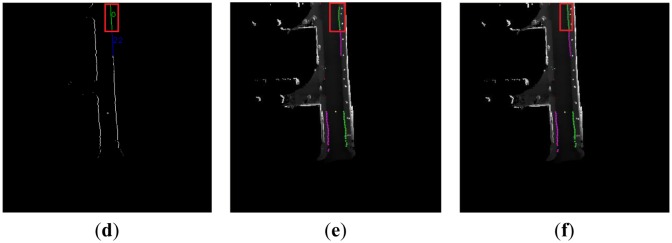
The contrastive result of the curved curb detection. (**a**) Data cluster result in Scene 5. (**b**) The bad curb detection result. (**c**) The result of our method. (**d**) Data cluster result in Scene 6. (**e**) The bad curb detection result. (**f**) The result of our method.

In [10], the authors used a cubic polynomial model to represent the curved curb. In some cases, the cubic polynomial curve is unsuitable, that is to say the model cannot fit the curved curb well. Compared with the above methods, we select the quadratic polynomial model and cubic polynomial model to represent the curved curb in the multi-model RANSAC algorithm. The contrastive results of the curved curb detection are shown in Figure 15, and results of the first row and the second row are detected in Scene 5 and Scene 6 respectively.

The results of the data cluster, bad detection and our algorithm are shown in the first, second and third column respectively. The red rectangle region denotes the same cluster in the same row. We use the quadratic polynomial model to obtain the result in Figure 15(b). Compared with Figure 15(a), the result lost the bottom data in red rectangle region in Figure 15(b). In Figure 15(e), the cubic polynomial model which is used in [10] obtains a bad result which missed the upper data in red rectangle region, but our algorithm can obtain the best curved curb in Figure 15(c,f). In short, our algorithm can select a better curved curb model than the algorithm in [10], and obtain good results.

**Figure 16.**
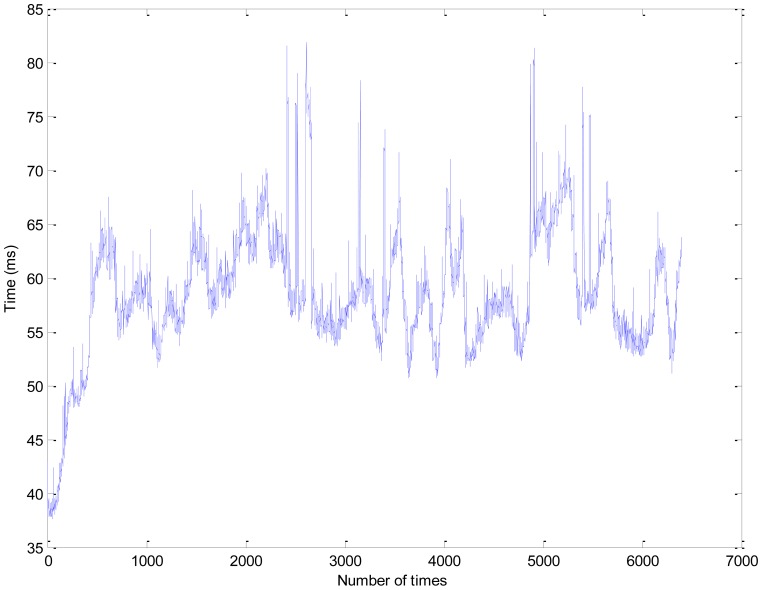
The execution time of our algorithm.

In the second experiment, the travelled distance of the vehicle is 3.2 km. The hardware and software specification in our system is shown in Table 1. The execution time of our algorithm in the second experiment is shown in Figure 16.

**Figure 17.**
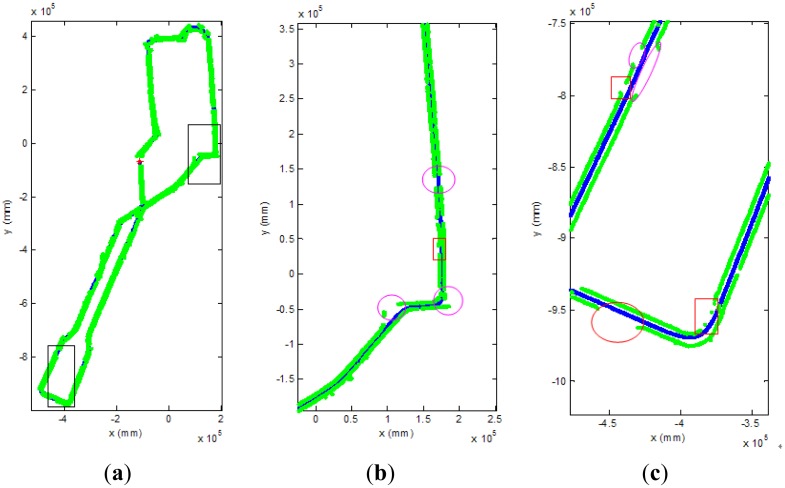
The curb detection results in the second experiment. (**a**) The entire curb detection results in the global coordinate system. (**b**) The enlarged result in the top rectangle in Figure 17(a). (**c**) The enlarged result in the bottom rectangle in Figure 17(a).

The average and the worst computational time of our algorithm are about 58.5 ms and 81.9 ms, respectively. In Figure 17, the curb detection results and the route of the vehicle is shown in the global coordinate system. Figure 17(a) is the entire curb detection results in the second experiment. The top and bottom black rectangle areas are enlarged in Figure 17(b,c), respectively. The blue lines denote the route of the vehicle, which are recorded from the navigation system of our vehicle, and the green points denote the detected curb points. The red rectangles and the purple ellipses denote the lost curb areas and the no curb areas which usually are the intersections on the road in Figure 17(b,c). The confusion matrix of the second experiment is shown in Table 2, and the data of the ground truth are labeled manually. Base on it, the true positive ratio and the true negative ratio are 86.8% and 93.4%. The accuracy of our algorithm is 87.8%. There are two reasons to the lost and wrong curb detection results. Firstly, the road structure is complex in our campus. There are many small intersections. Secondly, the curb candidate detection will lost some curb points, because the height of the curb changes a lot in different place. Thirdly, the laser data will miss in the water hole area on the road.

## 4. Conclusions

In this paper, a new curb detection method has been developed based on a local DEM which can be established with 2D sequential laser data and vehicle state data. The robustness and efficiency of the method have been demonstrated through various experiments. According to the experimental results of the four scenes, the proposed algorithm can not only detect road curbs in a static environment, but also in a typical dynamic environment.

Future research will focus on the fusion of camera and laser range finder data to extract the road surface, and on extending the curb information with recognized and classified obstacles and obstacle-free areas on the road.

## Figures and Tables

**Table 1.** The hardware and software specification.

**CPU**	**Intel(R) Core2 P8600 2.4 GHz**
Memory(RAM)	2 GB
Operating system	Windows XP Professional SP2
Programming language	C++

**Table 2.** The confusion matrix.

	**Predicted curb**	**Predicted no curb**
Actual curb	23248	3574
Actual no curb	341	4837
